# Supplementary material for: An organelle-specific protein landscape identifies novel diseases and molecular mechanisms
Source: Nat Commun. 2016 May 13;7:11491. doi: 10.1038/ncomms11491 (PMC4869170; doi:10.1038/ncomms11491)
Supplement: Supplementary Figures — 1-13 [file ncomms11491-s1.pdf]

Supplementary Figures

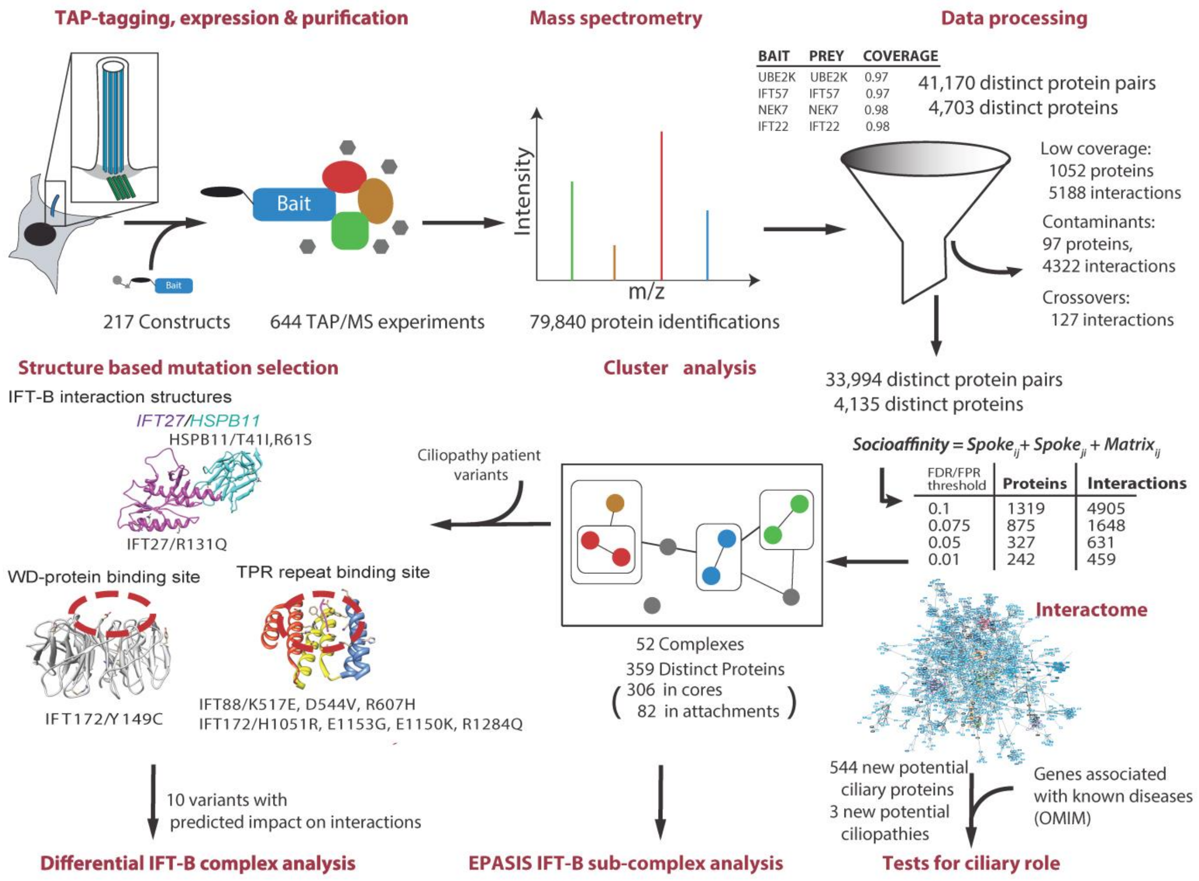

Supplementary Figure 1. Landscape identification strategy

The flow of data ranging from the original 217 selected constructs through expression, mass-spectrometry and data processing to arrive at interactions and complexes that are then investigated using a variety of computational and experimental techniques.

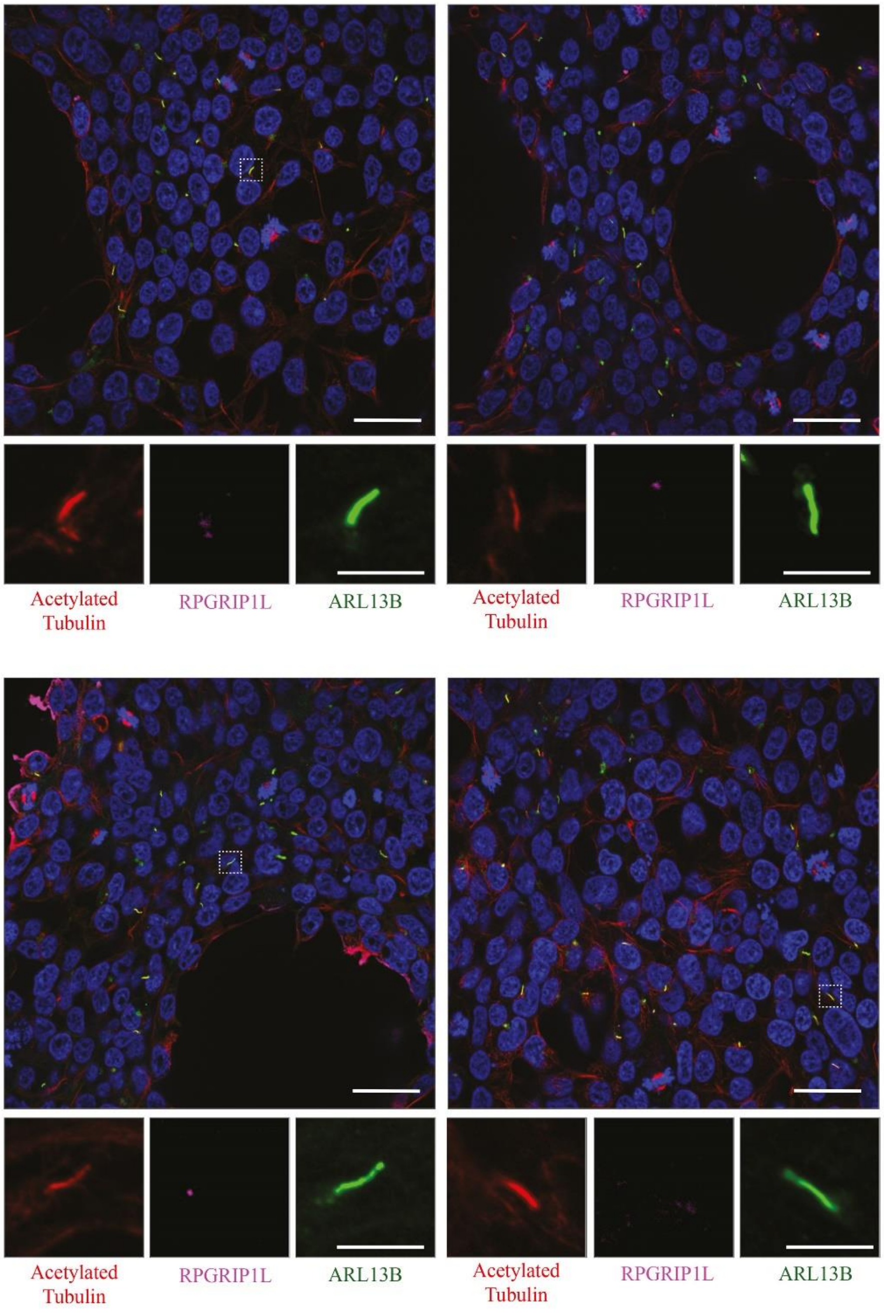

Supplementary Figure 2. HEK293-T cells are ciliated

HEK293T cells (passage number 10) were starved for 48 hours in 0,1% FCS starvation medium to enhance ciliogenesis prior to immunofluorescence staining of cells. Cells were stained with the ciliary marker ARL13B (green), the transition zone marker RPGRIP1L (pink), and the axonemal marker acetylated alpha-tubulin (red). Scale bar represents 20 µm. In the magnifications the scale bar represents 5 µm.

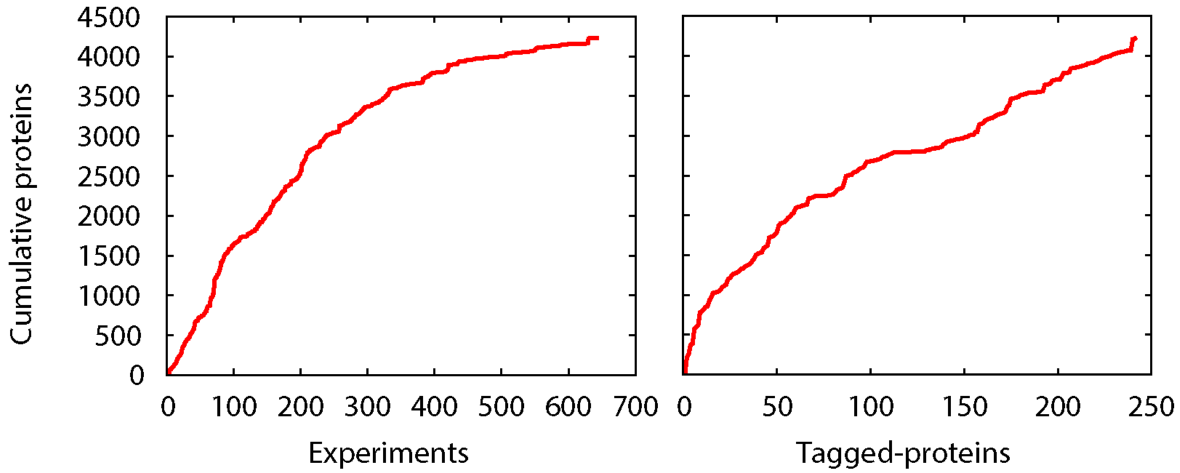

Supplementary Figure 3. Protein saturation curves

Plots showing the cumulative number of unique proteins identified as a function of the total number of experiments (left) and tagged-proteins (right). Note how the number of proteins gained diminishes as experiments progressed.

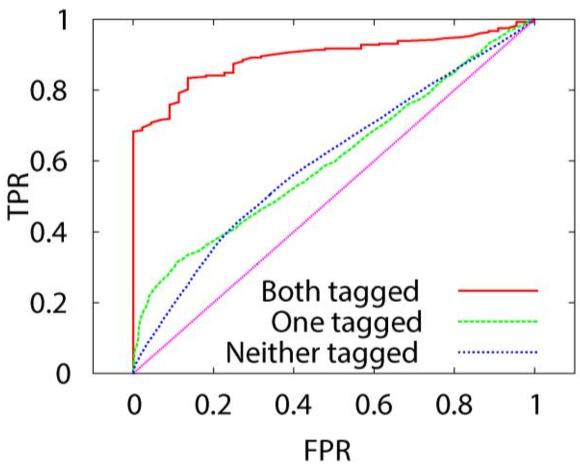

Supplementary Figure 4. ROC analysis showing how well Socioaffinity finds known interactions

Receiver operator characteristic (ROC) curve showing true-positive rate (TPR) versus false-positive rate (FPR) where socioaffinity thresholds were systematically lowered. The data are divided into three categories depending on whether both proteins, one protein or neither protein from each interacting pair was tagged in the experiment. Note the better performance of the reverse tagging (both tagged) relative to the others.

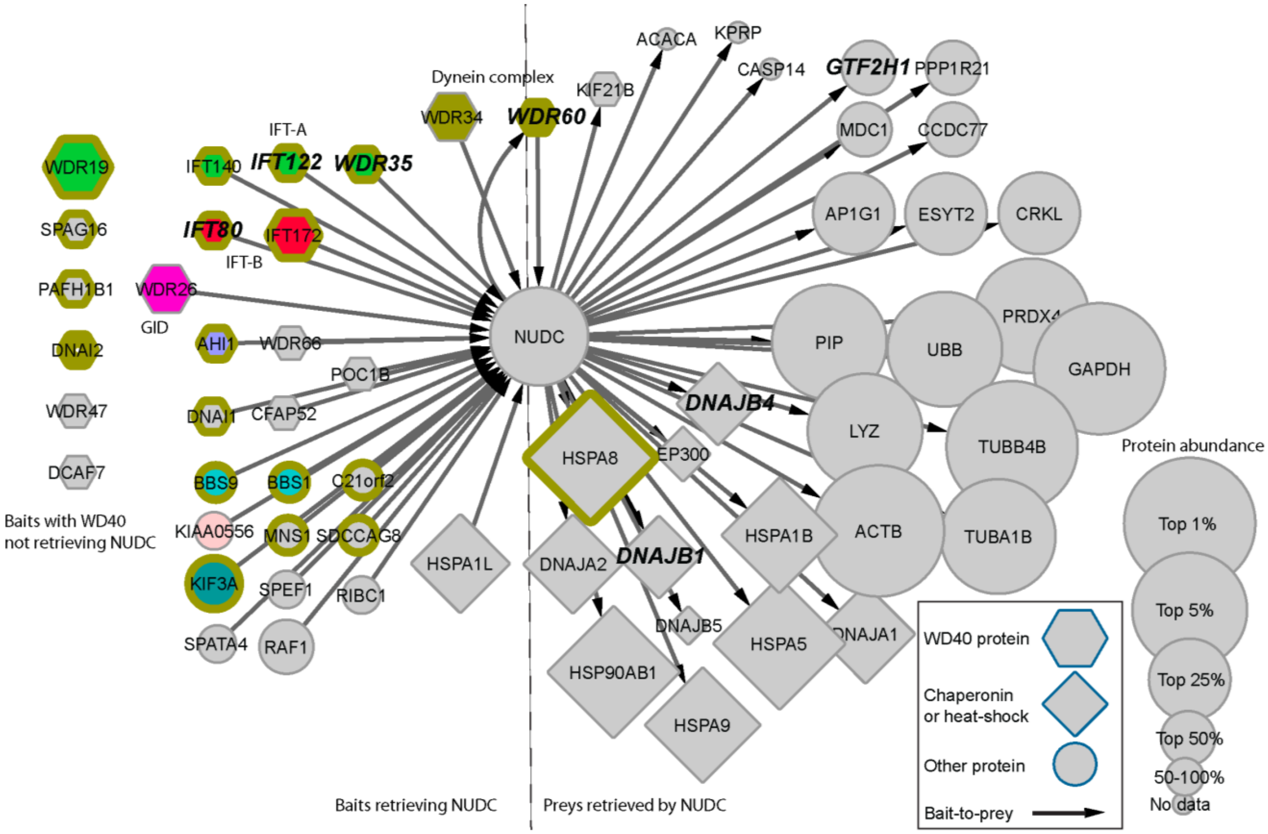

Supplementary Figure 5. Proteins identifying or identified by NUDC

Network showing proteins that identified NUDC when tagged (left) or retrieved with NUDC was tagged (right). Shapes denote different protein classes, and the size of each protein reflects its overall abundance as deduced by averaged protein counts from PaxDB in terms of where they were in the ranks of all human proteins. Proteins with gold boundaries are those from the Syscilia gold standard and those within our protein complexes are shaded other than grey. Protein labels in larger, bold, italicised text are those that had significant socioaffinities with NUDC in the final landscape.

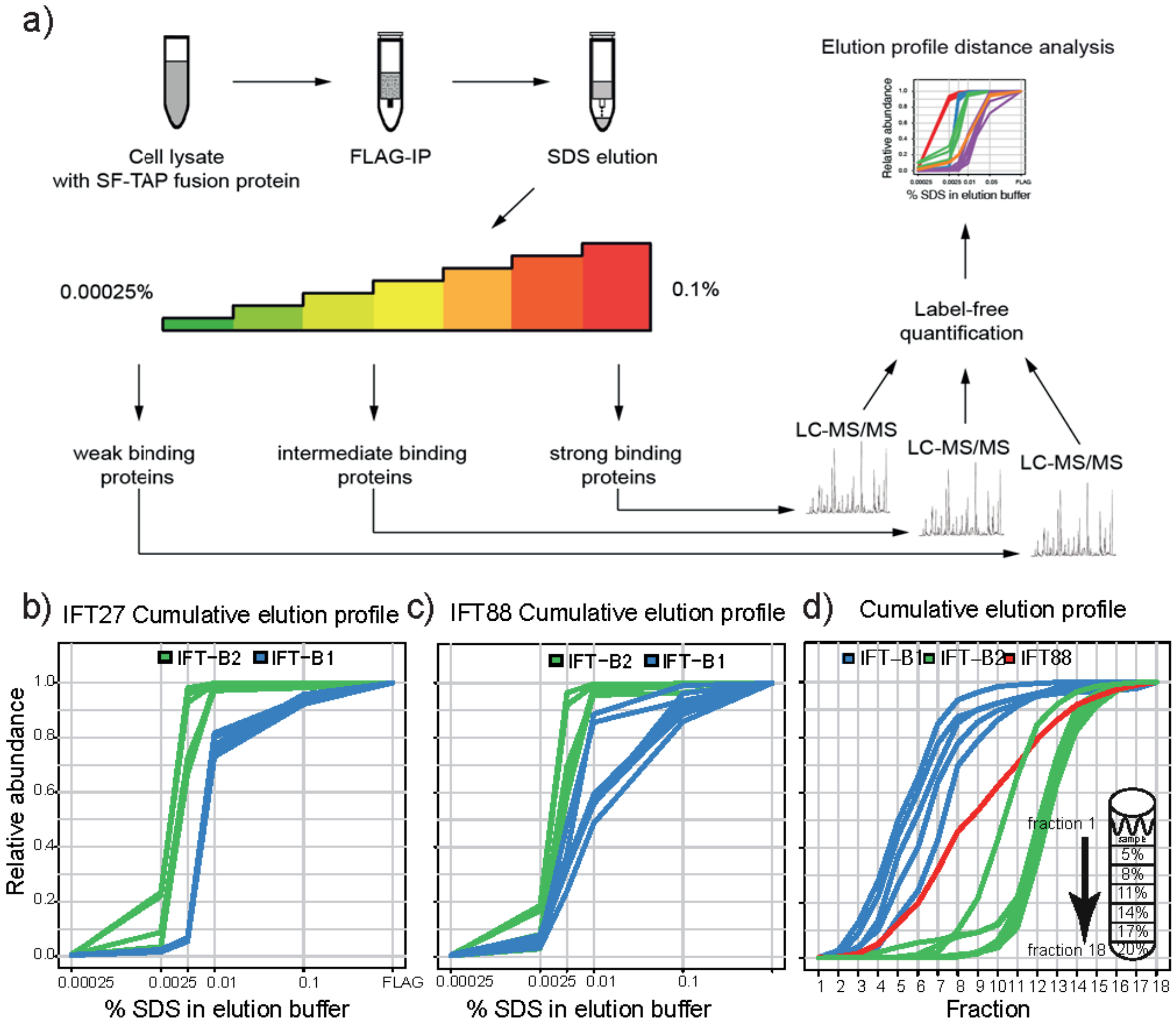

Supplementary Figure 6. Sub-complex analysis of IFT-B

a) Overview of the EPASIS approach for studying sub-complexes by TAP/MS. **b)** The IFT27 complex, purified from HEK293T cells, stably expressing IFT27-SF-TAP was isolated by FLAG purification and analyzed by EPASIS. The figure shows the cumulative elution profile of IFT-B1 and IFT-B2 complex components. **c)** The IFT88 complex, purified from HEK293T cells, stably expressing IFT88-SF-TAP was isolated by FLAG purification and analyzed by EPASIS. The figure shows the cumulative elution profile of IFT-B1 and IFT-B2 complex components. **d)** The IFT88 complex, purified from HEK293T cells, stably expressing IFT88-SFTAP was isolated by SF-TAP and subjected to sucrose density centrifugation to separate the IFT-B sub-complexes. The fractions were quantified by label-free mass spectrometry. The figure shows the cumulative elution profile of IFT-B1 and IFT-B2 complex components.

a)

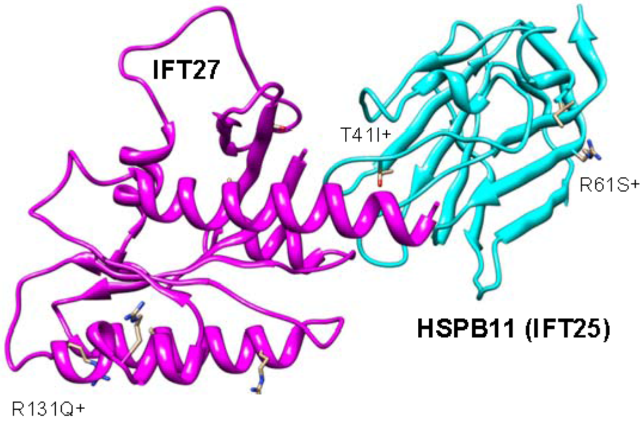

IFT27
T41I+
R61S+
HSPB11 (IFT25)
R131Q+

b)

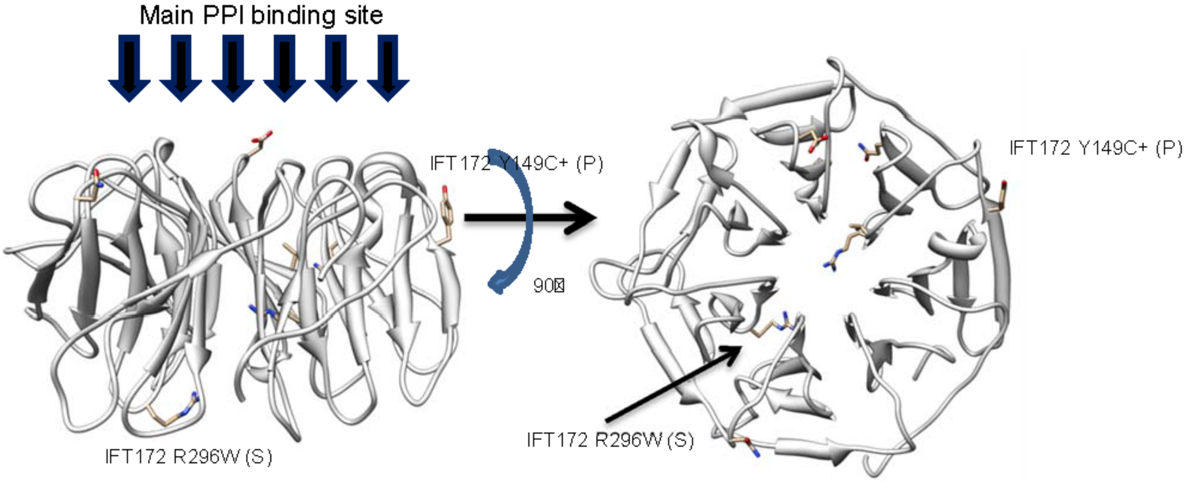

Main PPI binding site
IFT172 Y149C+ (P)
90
IFT172 Y149C+ (P)
IFT172 R296W (S)
IFT172 R296W (S)

c)

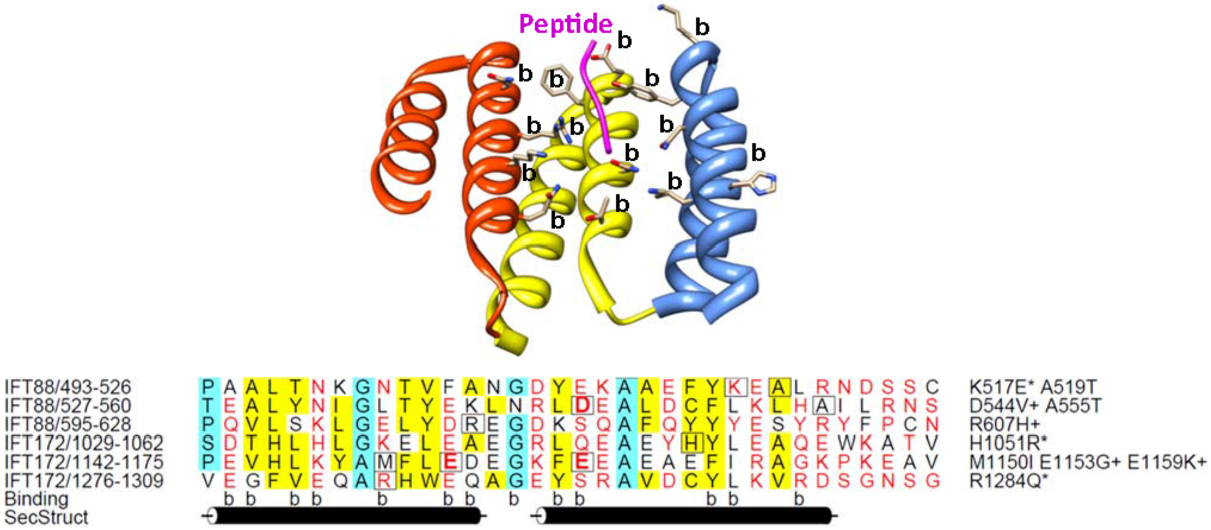

Peptide
b
IFT88/493-526 P A A L T N K G N T V F A N G D Y E K A A E F Y K E A L R N D S S C K517E* A519T
IFT88/527-560 T E A L Y N I G L T Y E K L N R L D E A L D C F L K L H A I L R N S D544V+ A555T
IFT88/595-628 P Q V L S K L G E L Y D R E G D K S Q A F Q Y Y Y E S Y R Y F P C N R607H+
IFT172/1029-1062 S D T H L H L G K E L E A E G R L Q E A E Y H Y L E A Q E W K A T V H1051R*
IFT172/1142-1175 P E V H L K Y A M F L E D E G K F E E A E A E F I R A G K P K E A V M1150I E1153G+ E1159K+
IFT172/1276-1309 V E G F V E Q A R H W E Q A G E Y S R A V D C Y L K V R D S G N S G R1284Q*
Binding b b b b b b b b b b b b b
SecStruct

Supplementary Figure 7. Structure based selection of ciliopathy variants affecting protein interactions

a) Structures of the (left) IFT27 (magenta) / HSPB11 (cyan) interactions between orthologues from *Chlamydomonas reinhardtii* (left) with the location of variants identified in ciliopathy cases marked. Those selected as having the potential to affect interactions are marked with a plus (+) symbol. Structures are taken from RCSB/PDB codes 2YC4 and 4UZY respectively. **b)** Two views of the structure of a WD40 domain showing the location of human variants in various IFT proteins and the location of the most common protein binding site in this protein family. Variants are marked as protein-binding site (P), core (C) or surface (S) and those selected as candidates to affect interactions are marked with a plus (+) symbol. The structure is coloured blue-yellow-red to reflect the approximate N- C- terminal direction of the polypeptide. **c)** Structure (left) of a TPR repeat showing the location of disease variants in various IFT proteins and the location of the most common protein binding site residue positions (b) for this family of protein repeats. Alignment (right) showing the location of the same variants (boxed). The alignment is coloured according to the most prominent residue properties at each position: yellow background, hydrophobic; blue background, small; red characters, polar. The 'b's at the bottom of the figure denote the position of the common binding site residues, above the location of the alpha helices.

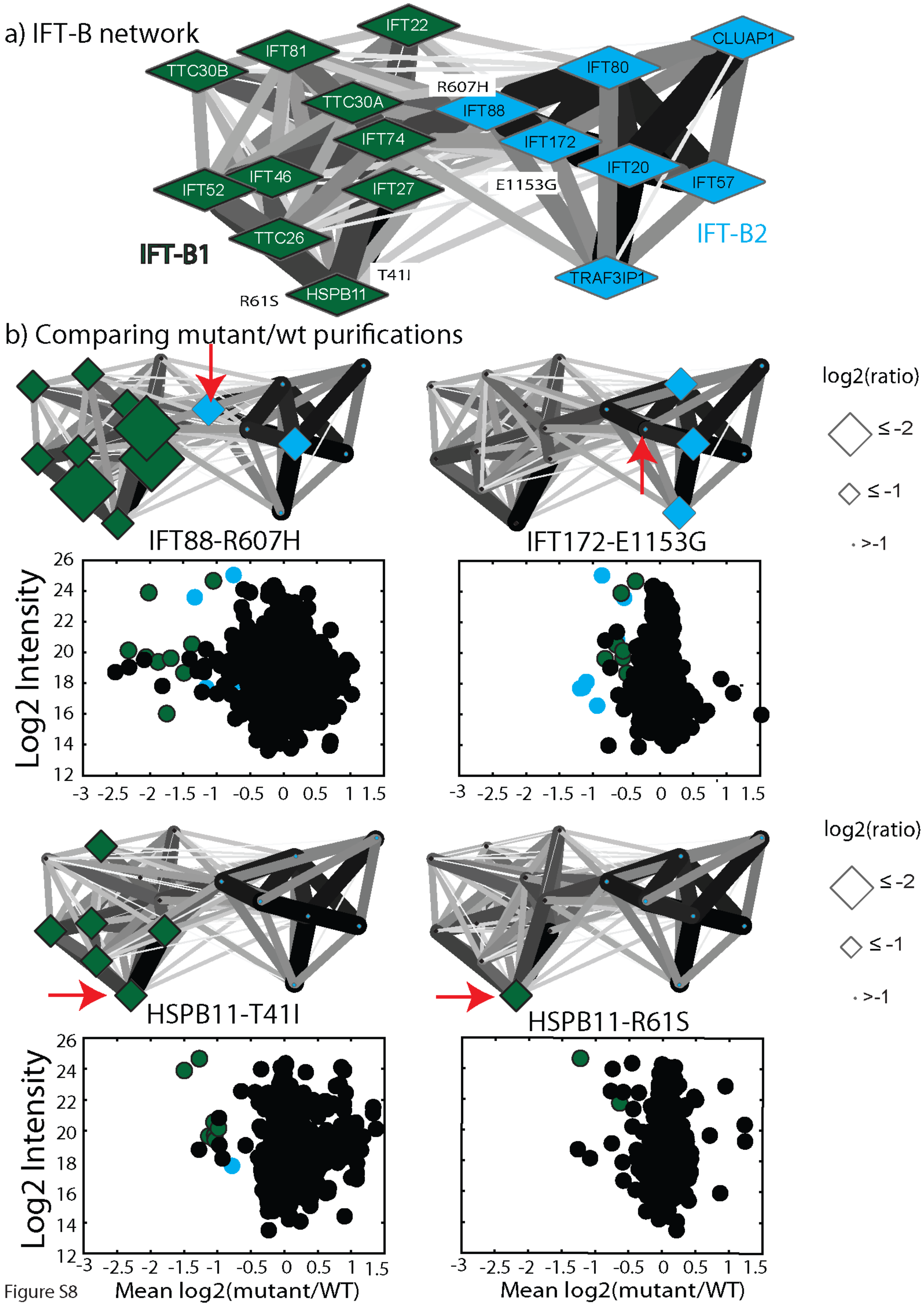
a) IFT-B network
IFT22
IFT81
TTC30B
CLUAP1
IFT80
R607H
TTC30A
IFT88
IFT74
IFT172
IFT46
IFT20
IFT52
IFT27
E1153G
IFT57
TTC26
IFT-B1
IFT-B2
T41I
TRAF3IP1
R61S
HSPB11
b) Comparing mutant/wt purifications
log2(ratio)
≤ -2
≤ -1
>-1
IFT88-R607H
IFT172-E1153G
Log2 Intensity
HSPB11-T41I
HSPB11-R61S
Mean log2(mutant/WT)

Figure S8

Supplementary Figure 8. How disease variants affect IFT-B subcomplexes

a) Network of IFT-B (top) drawn using a Socioaffinity-weighted spring embedded automatic layout (in Cytoscape) with the two sub-complexed coloured green (IFT-B1/core) or orange (IFT-B2/periphery). **b)** Network representation (layout as for a) of protein depletions within IFT-B sub-complexes as revealed by differential TAP-MS analysis comparing mutant to wild-type. The plots below each network are of Log_2 intensity versus the ratio of a tagged mutant versus a tagged wild-type with values below 0 denoting proteins that were lower in the mutant relative to the wild-type. Proteins in each subunit are coloured as for the top of the figure. Green nodes represent proteins of the IFT-B1, blue nodes represent proteins of the IFT-B2 complex.

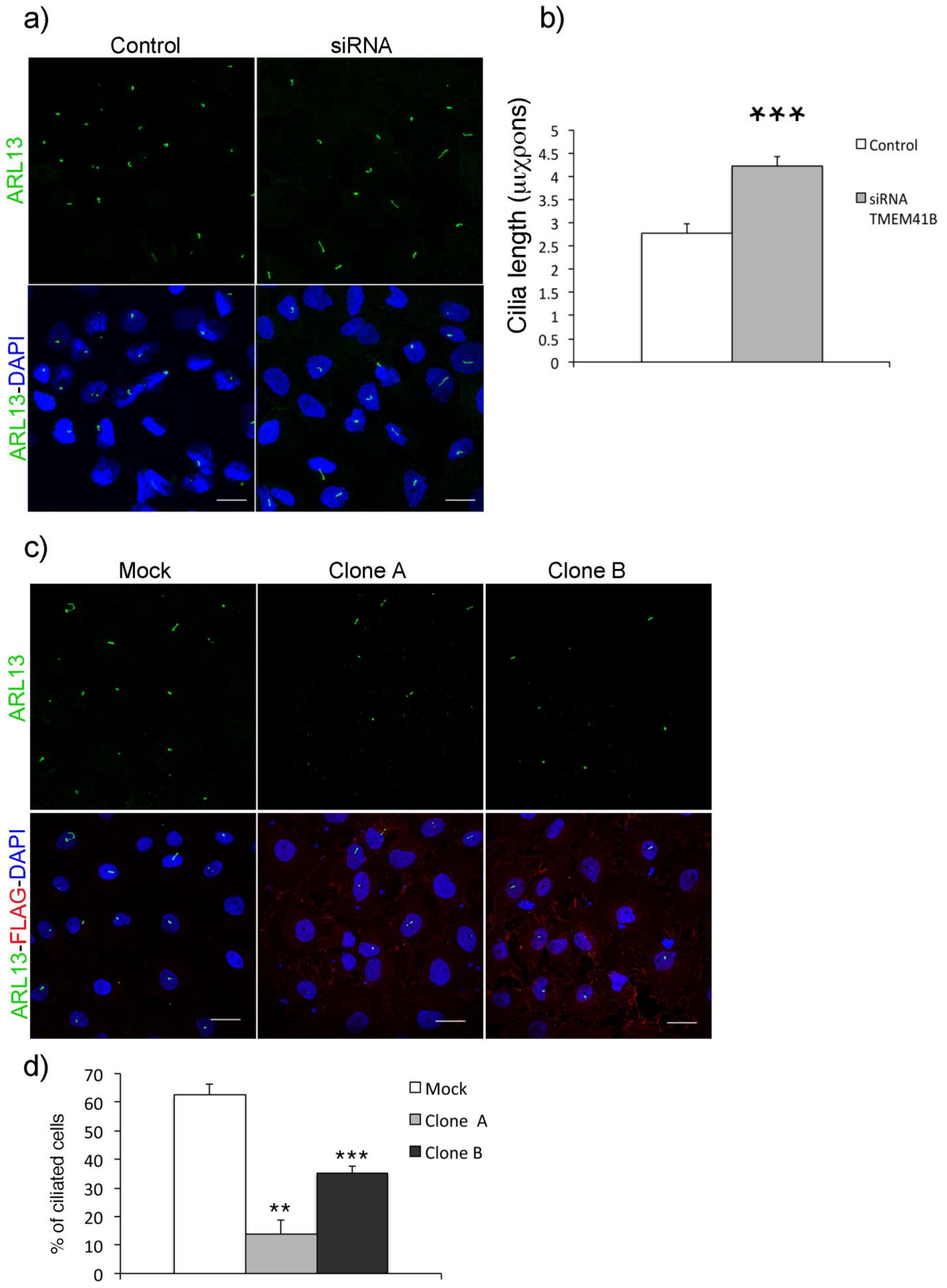
a)
Control
siRNA
ARL13
ARL13-DAPI
b)
***
Cilia length (μχρons)
5
4.5
4
3.5
3
2.5
2
1.5
1
0.5
0
Control
siRNA TMEM41B
c)
Mock
Clone A
Clone B
ARL13
ARL13-FLAG-DAPI
d)
% of ciliated cells
70
60
50
40
30
20
10
0
**
***
Mock
Clone A
Clone B

Supplementary Figure 9. TMEM41B negatively regulates the length of primary cilia

a) HK2 cells were treated with control siRNA or siRNA targeting TMEM41B respectively, serum starved for 24h, and stained for ARL13B (green) and DNA (blue). **b)** Ciliary length was measured in ciliated cells based on ARL13B staining. **c)** HK2 cells were transiently transfected with a vector expressing p3XFLAG (red), clone A and clone B respectively, serum starved for 24h, and were stained with ARL13B (green) and DNA (blue). **d)** Percentage of ciliated cells was represented in the graphic. a) and c) Scale bars represent 10μm. b) and d) biological triplicates were analysed and a t-test was performed. p-values below 0.01 are represented by ** and below 0.001 by ***. Error bars represent the s.e.m.

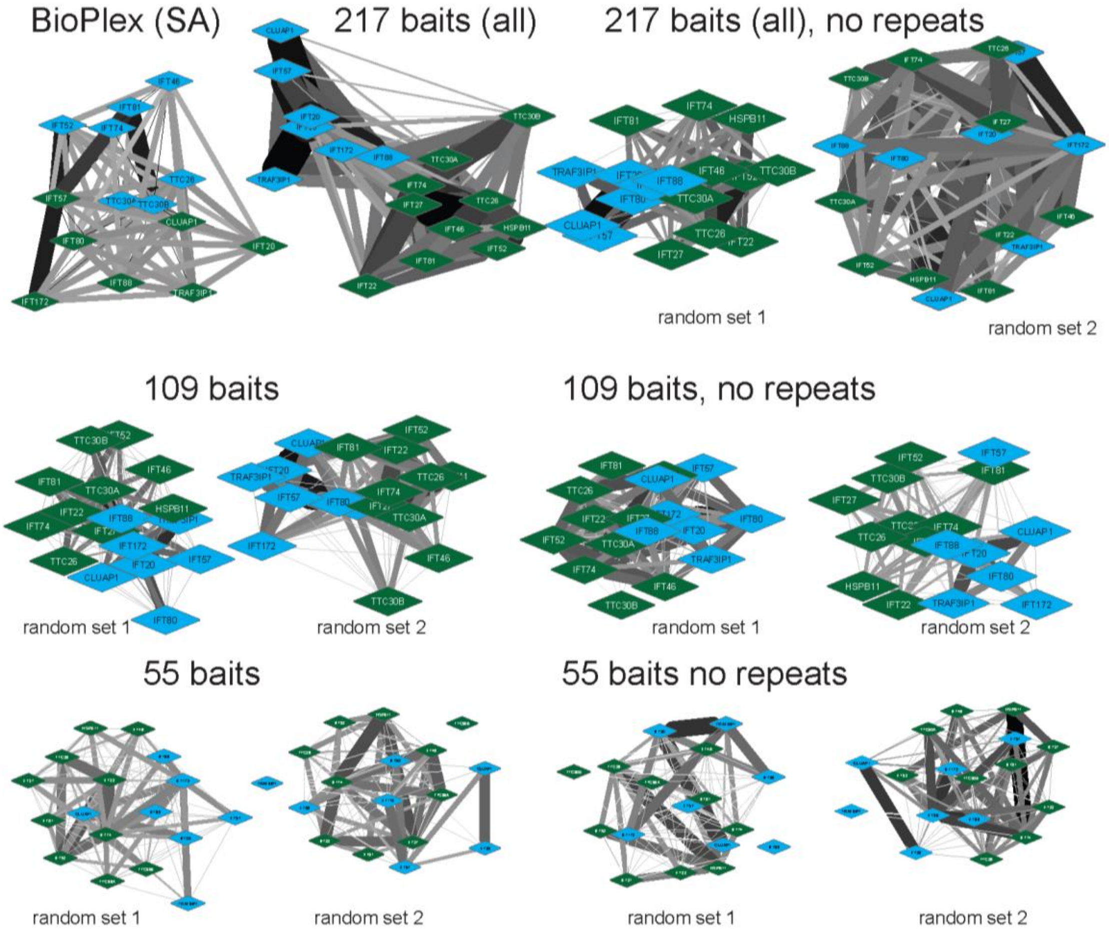

Supplementary Figure 10. Loss of resolution in IFT-B sub-structure with reduced TAP-MS datasets

Illustration of how sub-complex architecture diminishes with less complete datasets. ‘Bioplex (socioaffinity)’ shows the network derived for IFT-B from the recently published human TAP dataset. ‘217 baits’ shows the complex derived from our data. Networks to the right and below show the effect of removing parts of the dataset, either by skipping the repeats (‘no repeats’) or considering only half (109) or a quarter (55) of the baits (or both). We generated random sets multiple times and got different results, illustrating a ‘luck of the draw’ effect both with regard to which baits and interestingly with regard to which repetition of each bait was taken (these are illustrated twice for each simulated scenario and labelled ‘random set 1’ or ‘random set 2’. All networks are generated socioaffinity-weighted spring embedded automatic layout (Cytoscape). Green nodes represent proteins of the IFT-B1, blue nodes represent proteins of the IFT-B2 complex.

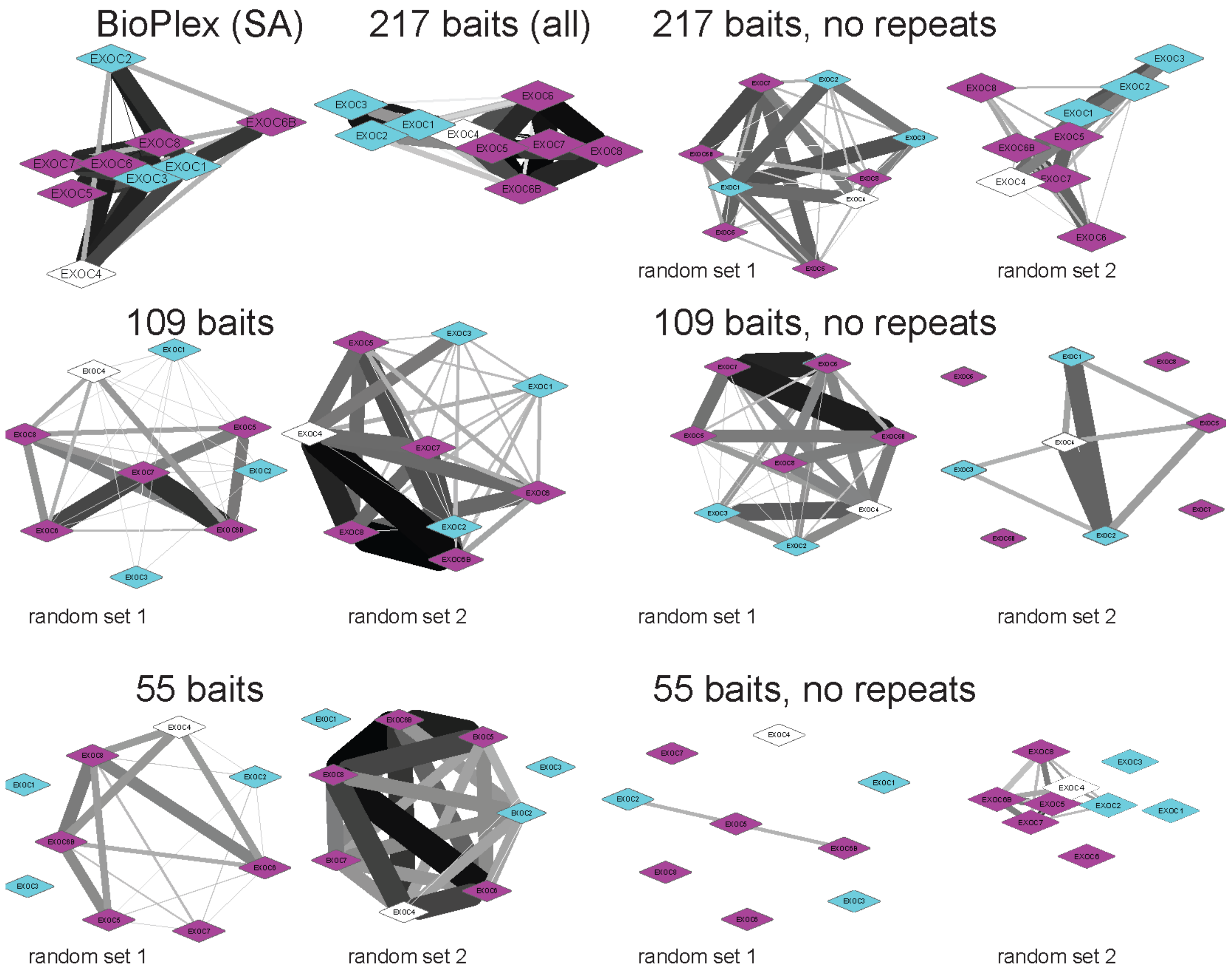

Supplementary Figure 11. Loss of resolution in Exocyst sub-structure with reduced TAP-MS datasets

As for Fig. S10, but considering the Exocyst complex in place of IFT-B. Purple nodes represent components of the Exocyst sub-complex 1, blue nodes represent components of the Exocyst sub-complex 2.

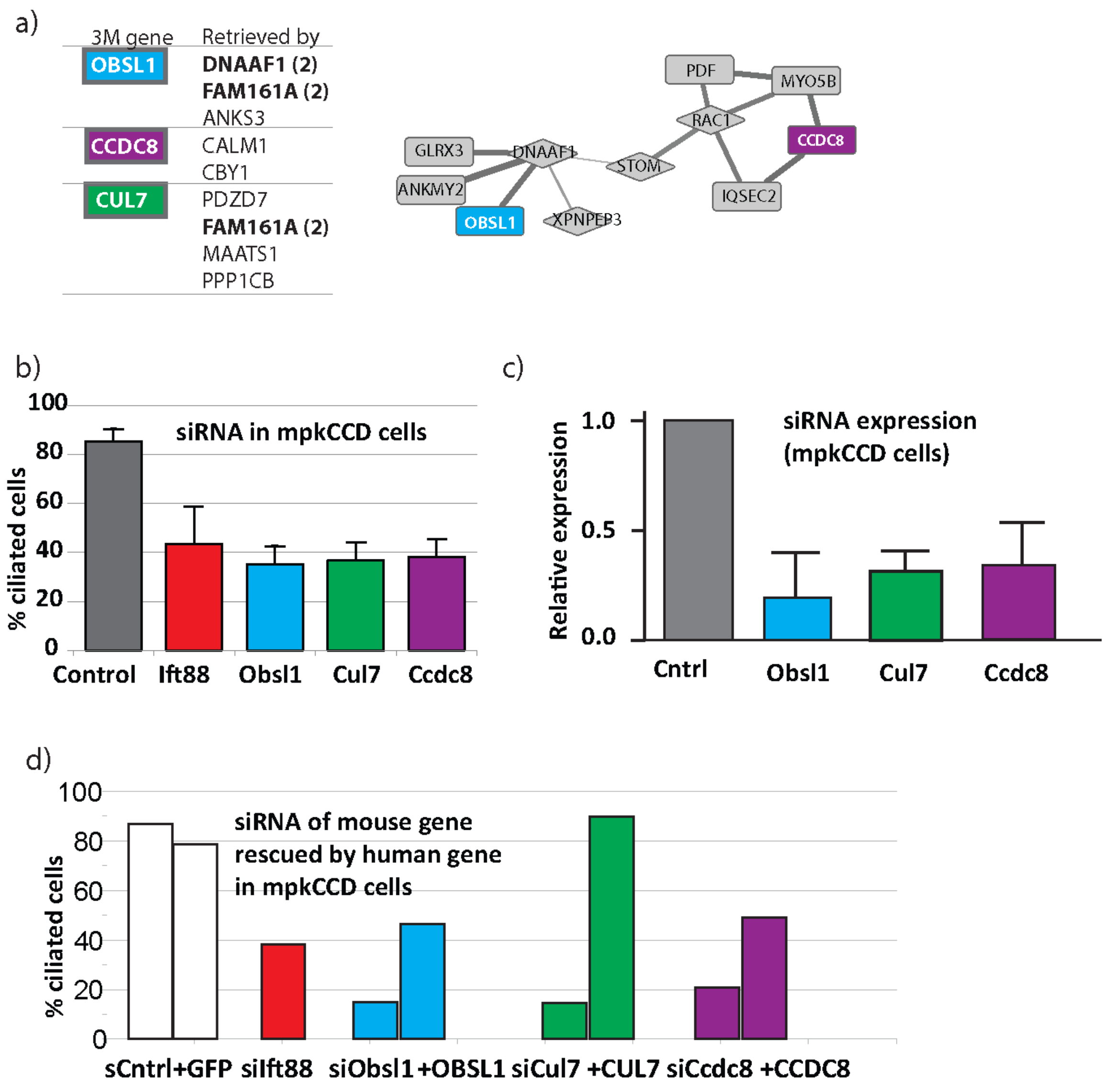

Supplementary Figure 12. siRNA shows all three 3M genes have a ciliary phenotype

a) Overview of purifications retrieving 3M complex genes and socioaffinity network as shown in Fig. 5 (for convenience). b) Effect of siRNA for selected genes in 3M (and Ift88 for comparison to a known ciliary gene) on the number of ciliated mpkCCD cells observed. c) Relative expression of 3M genes studied in b) showing a loss of expression relative to control d) Rescue of ciliary phenotype through transient overexpression of human orthologs of the silenced mouse 3M genes. Mouse and human genes are labelled with standard capitalization (e.g. Obsl1 in mouse has OBSL1 in human as an ortholog). b)-c) For all experiments biological triplicates with technical duplicates were performed. Error bars represent the s.e.m.

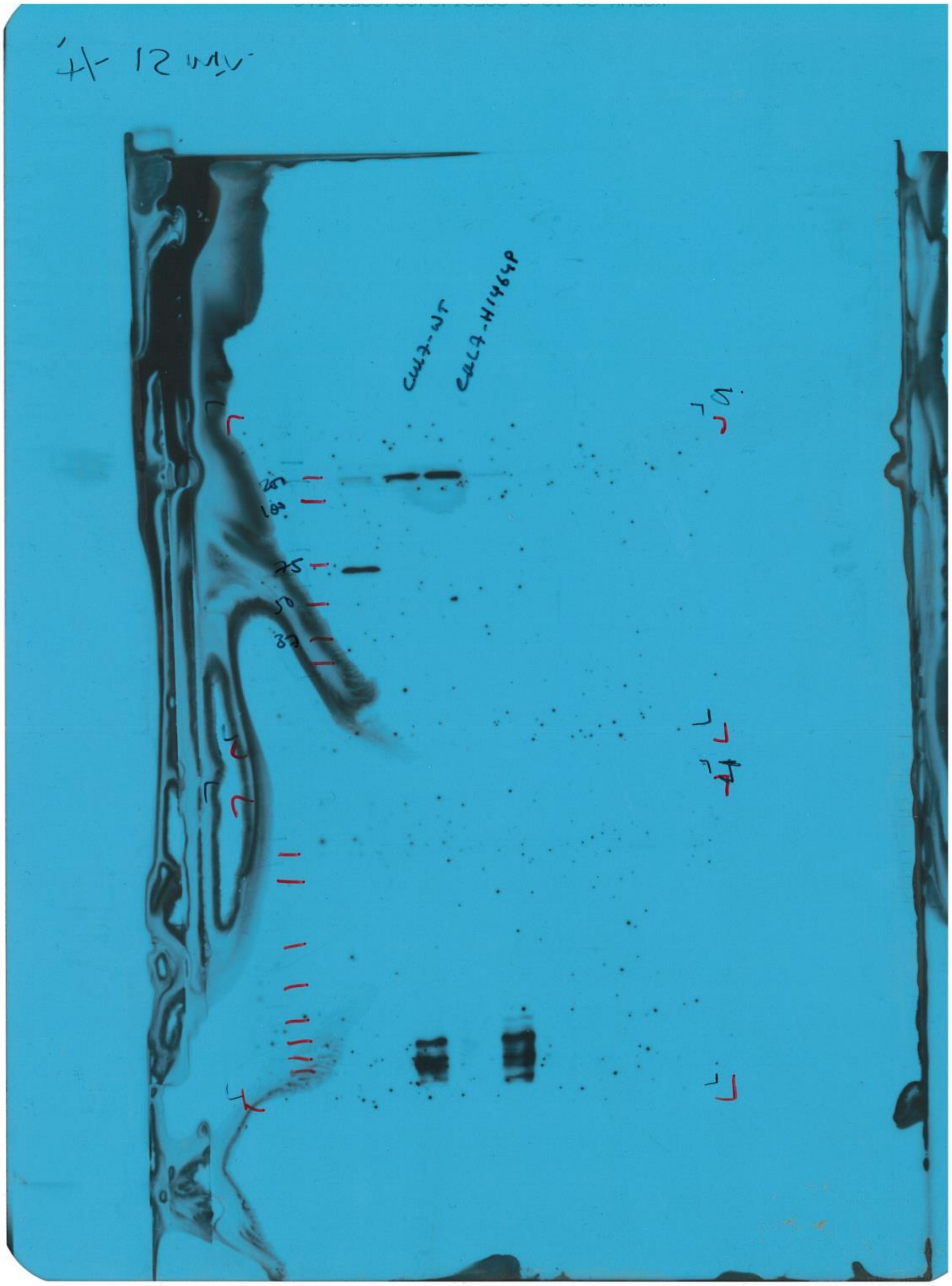

Supplementary Figure 13. Patient 3M protein CUL7 p.H1464P is expressed

Western blot showing that the mutated patient protein is expressed to a level similar to wild-type in cultured fibroblasts, indicating that the lack of rescue in ciliary frequency is not due to lack of expression, but loss of function.
